# A TiO_2_@MWCNTs nanocomposite photoanode for solar-driven water splitting

**DOI:** 10.3762/bjnano.13.125

**Published:** 2022-12-14

**Authors:** Anh Quynh Huu Le, Ngoc Nhu Thi Nguyen, Hai Duy Tran, Van-Huy Nguyen, Le-Hai Tran

**Affiliations:** 1 Ho Chi Minh City University of Natural Resource and Environment, 236B Le Van Sy street, Tan Binh District, Ho Chi Minh City, Vietnam; 2 Faculty of Chemical Engineering, Ho Chi Minh City University of Technology (HCMUT), 268 Ly Thuong Kiet street, District 10, Ho Chi Minh City, Vietnam; 3 Vietnam National University Ho Chi Minh City, Linh Trung Ward, Thu Duc City, Ho Chi Minh City, Vietnam; 4 Faculty of Allied Health Sciences, Chettinad Hospital and Research Institute, Chettinad Academy of Research and Education, Kelambakkam-603103, Tamil Nadu, India

**Keywords:** multi-wall carbon nanotubes (MWCNTs), nanomaterials, photoelectrochemical, TiO_2_, water splitting

## Abstract

A TiO_2_@MWCNTs (multi-wall carbon nanotubes) nanocomposite photoanode is prepared for photoelectrochemical water splitting in this study. The physical and photoelectrochemical properties of the photoanode are characterized using field emission-scanning electron microscopy, transmission electron microscopy, X-ray diffraction, and linear sweep voltammetry. The results show that the TiO_2_@MWCNTs nanocomposite has an optical bandgap of 2.5 eV, which is a significant improvement in visible-light absorption capability compared to TiO_2_ (3.14 eV). The cyclic voltammograms show that incorporating TiO_2_ with the MWCNTs leads to a decrease in the electrical double layer, thereby facilitating the electron transfer rate in the TiO_2_@MWCNTs electrode. Moreover, the current density of the photoelectrochemical electrode formed by TiO_2_@MWCNTs under solar irradiation is significantly higher than that prepared by TiO_2_ (vs Ag/AgCl). The low charge capacity of the TiO_2_@MWCNTs electrode–electrolyte interface hinders the recombination of the photogenerated electrons and holes, which contributes to the enhancement of the solar-to-hydrogen (STH) conversion efficiency. The average STH conversion efficiency of the TiO_2_@MWCNTs electrode under solar exposure from 6 AM to 5 PM is 11.1%, 8.88 times higher than that of a TiO_2_ electrode. The findings suggested TiO_2_@MWCNTs is a feasible nanomaterial to fabricate the photoanode using photoelectrochemical water splitting under solar irradiation.

## Introduction

TiO_2_ is an excellent photochemical catalyst for environmental and chemical applications due to its good activity regarding numerous reduction and oxidation reactions. As a wide-bandgap (ca. 3.2 eV) semiconductor, TiO_2_ is a promising photocatalyst for degrading a massive range of high-molecular-weight organic pollutants under UV radiation [1]. Because of high specific surface, nanoscale TiO_2_ as grains or tubes can absorb UV light more substantially than mesoscale TiO_2_ [2–3]. This results in an improvement of the photon efficiency of TiO_2_ nanoparticles. Reducing the dimension of the photocatalyst favors not only a bandgap shift to the visible-light region but, unfortunately, also the recombination of photogenerated electrons and holes (e^−^/h^+^), which limits the photocatalytic performance [4–5].

Because TiO_2_ only exhibits photochemical activity under UV excitation, which accounts for a small fraction (ca. 4%) of the solar energy, numerous modification methods such as doping with nonmetals, coupling with other catalysts, and attaching to supports have been developed to increase the absorption of visible solar light [6–7]. Notably, carbon nanotubes (CNTs) are a promising material for visible-light absorption [8]. A combination of TiO_2_ with CNTs can effectively enhance the separation of e^−^/h^+^ pairs based on the high electric conductivity of CNTs. This approach improves solar water splitting performance [7,9]. However, an excess amount of CNTs can deteriorate the photoactivity of TiO_2_ nanoparticles because CNTs block and cover the surface of TiO_2_ [9].

There are three categories of water splitting techniques applying photocatalysts, namely photocatalytic, photoelectrochemical, and photovoltaic–photoelectrochemical systems. The features and the operating mechanism of photoelectrochemical water splitting are detailed in [10–11]. Photoelectrochemical water splitting has attracted much research interest because it has some outstanding advantages. The research focuses on synthesizing and modifying photocatalysts for photoanodes and photocathodes for photoelectrochemical water splitting [11]. Several TiO_2_-based photocatalysts have been developed and applied in photoelectrochemical water splitting. The results showed that the solar-to-hydrogen (STH) conversion efficiency of TiO_2_-based photoanodes (0.2–0.42%) is lower than that of TiO_2_/CNT anodes (4.4%), which is attributed to a wider bandgap of the TiO_2_ photocatalyst and the lesser extent of e^−^/h^+^ pair recombination [12]. Dai et al. prepared a MWCNTs/TiO_2_ (MWCNTs = multi-wall carbon nanotubes) nanocomposite by sol–gel method for visible-light-induced photocatalytic hydrogen evolution [8]. The photocatalyst consisted of dense TiO_2_ particles covering functionalized MWNTs and exhibited good photoactivity under visible light (λ > 420 nm), but the photoelectrochemical water splitting showed a low hydrogen evolution of 450 µmol·h^−1^. Reddy et al. loaded TiO_2_ particles on MWCNTs via a simple hydrothermal method [13]. However, the MWNTs/TiO_2_ nanocomposite showed photoactivity only under UV irradiation due to the high bandgap of 3.1 eV.

To the best of our knowledge, there are only a few studies on TiO_2_@MWCNTs nanocomposites as photoanode material for photoelectrochemical water splitting. Furthermore, the preparation of TiO_2_/MWCNTs nanocomposite derived from TiO_2_ precursors and functionalized MWCMTs for photoelectrochemical water splitting is based on complicated and time-consuming hydrothermal and sol–gel methods. Herein, a TiO_2_@MWCNTs nanocomposite photocatalyst is synthesized via a simple hydrolysis method. The coupling of TiO_2_ and MWCNTs aims to limit the recombination of photogenerated electrons and holes, to improve the visible-light absorption of the photoanode under solar irradiation, and to enhance the hydrogen evolution. The morphology and photoelectrochemical properties of the TiO_2_@MWCNTs electrode are systematically studied, and the efficiency of the electrode in photoelectrochemical water splitting is also demonstrated.

## Experimental

### Materials

Multi-wall carbon nanotubes (purity >99.5%), synthesized via chemical vapor deposition were supplied by Vinanotech (Vietnam). Titanium tetrachloride (purity >99%) was purchased from Sigma-Aldrich (USA), and pure potassium hydroxide and potassium chloride (purity >85%) were provided from Merck (Germany). All other chemical reagents used in this study were of reagent grade.

### Preparation of photocatalyst and photoelectrode

#### TiO_2_ powders

TiO_2_ powders were simply prepared from a violent hydrolysis reaction of TiCl_4_ vapor in humid air. Precipitated fine TiO_2_ particles were collected before thermal treatment at 350 °C for 60 min.

#### TiO_2_@MWCNTs nanocomposite

First, 1.0 g of MWCNTs are added to 25 mL of TiCl_4_ in a glass beaker under an inert atmosphere. Following, MWCNTs are dispersed in TiCl_4_ under ultrasound for 15 min. After that, an excessive amount of deionized water is slowly dropped into the mixture. Then, the obtained solid phase is filtered and washed with water to neutralize. Finally, it is treated at 350 °C within 60 min to form the TiO_2_@MWCNTs nanocomposite photocatalyst.

#### Photoelectrochemical electrode

1.0 g of the obtained photocatalyst is dispersed in a 25 mL solution containing 0.1 M of polyaniline and 0.3 M of oxalic acid. A plastic bar (15 × 35 × 3 mm^3^ of width × height × thickness) as the support for the electrode is immersed in the as-prepared mixture. The catalyst mixture is assembled on the plastic bar under ultrasound for 1 min. Finally, the photoelectrochemical electrode is obtained after drying at 60 °C for 15 min.

### TiO_2_@MWCNTs nanocomposite characterizations

The surface morphology of MWCNTs and the TiO_2_@MWCNTs nanocomposite is characterized by using field-emission scanning electron microscopy (FE-SEM, S4800) and transmission electron microscopy (TEM, JEOL-1400). The crystallization behavior of the catalysts is analyzed by X-ray diffraction (XRD, D2 PHASER). The chemical structure of the samples is characterized using Fourier-transform infrared spectroscopy (FTIR, Brucker 27). The electrochemical measurements are carried out on a MPG2 Biologic system with a three-electrode cell controlled by ECLab® software. Diffuse reflectance spectra (DRS) are recorded with a spectrophotometer (FL-1039, HORIBA) using a 450 W xenon lamp. A portable Lux-meter (MW700, Milwaukee) and a pyranometer (SR30, Huseflux) are used for sunlight intensity and irradiance measurements.

### Photoelectrochemical measurements

Electrochemical measurements are performed in 0.1 M KCl solution at a scan rate of 50 mV·s^−1^ with a three-electrode cell using Ag/AgCl and Pt wire as the reference and counter electrodes, respectively. The prepared TiO_2_@MWCNTs electrode is used as the working electrode. The measurement is carried out in a lab under 350 ± 10 lux of naturally luminous emittance. The relation of current and potential is recorded under dark (D) and light (L) conditions corresponding to the sunlight intensity below 10 lux and around 100 lux.

**Figure 1:**
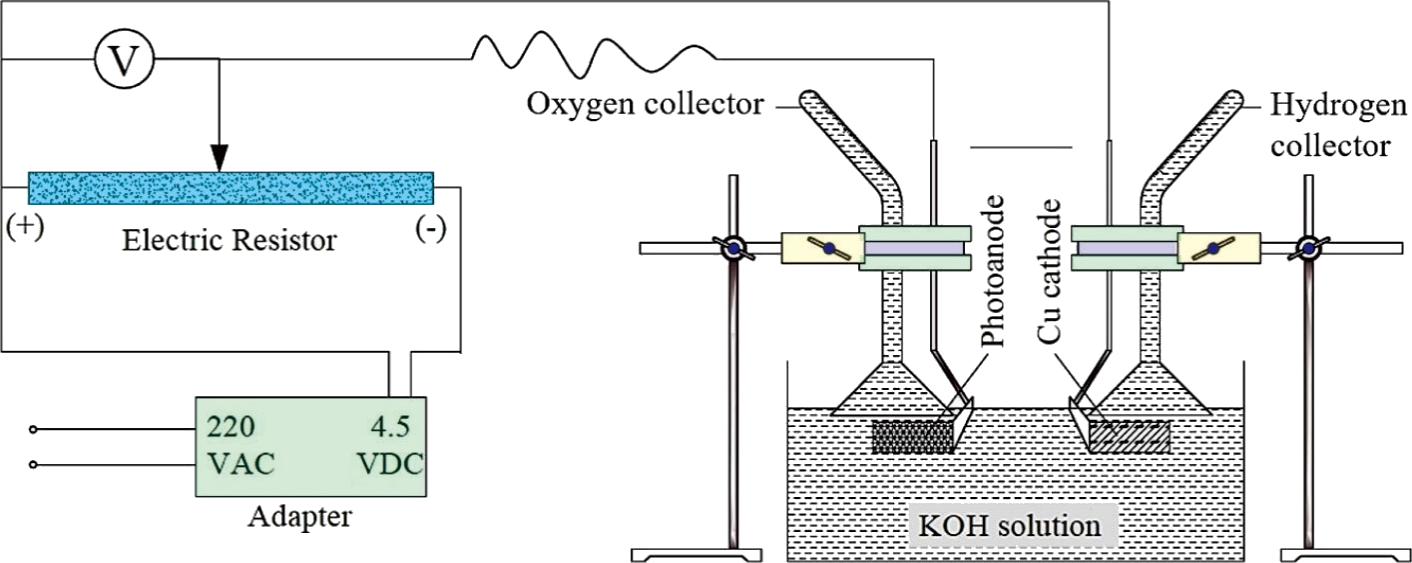
Schematic of the photoelectrochemical water splitting experimental apparatus.

Photoelectrochemical water splitting performance experiments are carried out under natural sunlight using a two-electrode cell, including the photoanode and a Cu-based cathode. Hydrogen evolution at the cathode and the solar irradiance is recorded at 60 min intervals. The prepared photoelectrochemical electrode is wholly immersed in KOH electrolyte before each photoelectrochemical measurement. Only one electrode surface with a size of 1.5 × 2.0 cm^2^ is irradiated. A schematic of the experimental apparatus is described in Figure 1.

## Results and Discussion

### Characterization of the TiO_2_@MWCNTs nanocomposite catalyst

**Figure 2:**
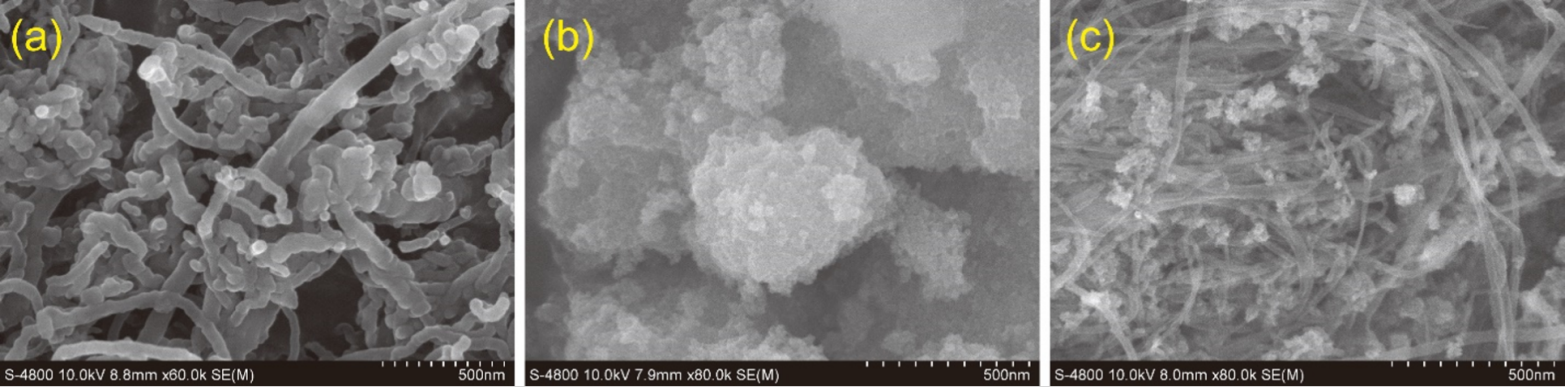
SEM images of (a) MWCNTs, (b) TiO_2_, and (c) the TiO_2_@MWCNTs nanocomposite.

FE-SEM images of the morphology of the MWCNTs, TiO_2_ powder, and the TiO_2_@MWCNTs nanocomposite are shown in Figure 2. They confirm that the TiO_2_@MWCNTs nanocomposite catalyst is successfully prepared via the hydrolysis reaction. It can be seen from Figure 2a that the morphology of MWCNTs is incoherent, curved, interlaced, and with little branching. Furthermore, a non-uniform decoration of TiO_2_ clusters is observed on the surface of the MWCNTs (Figure 2c).

**Figure 3:**
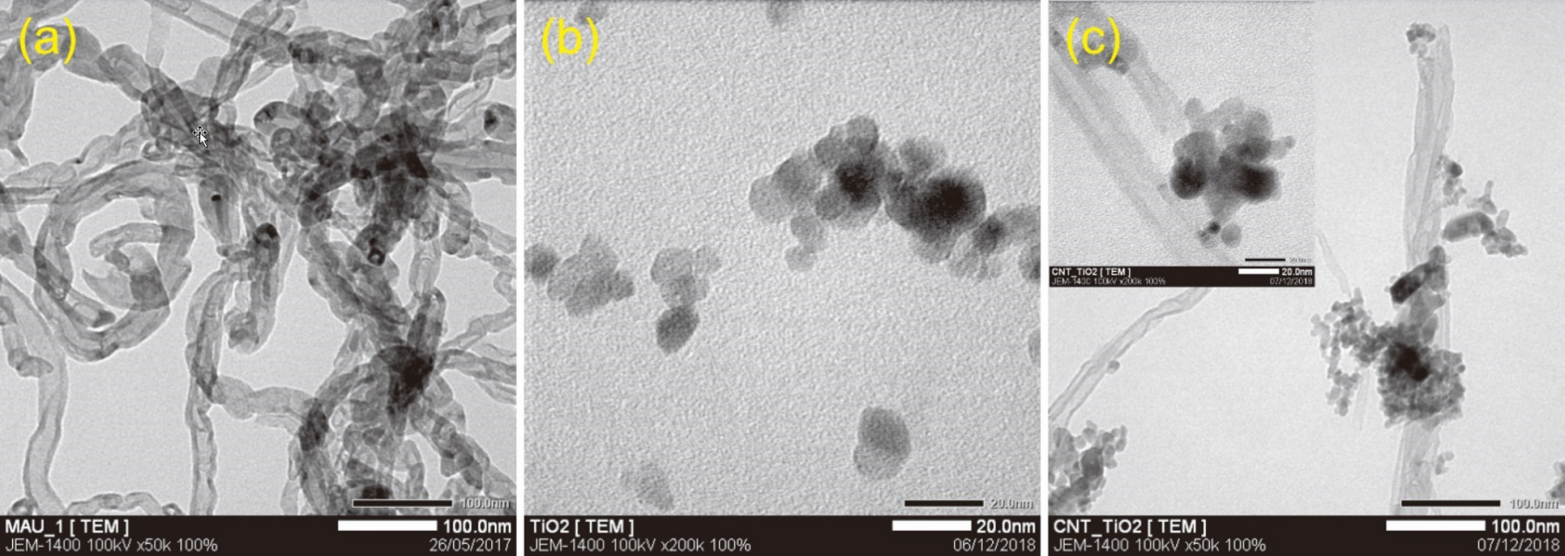
TEM images of (a) MWCNTs, (b) TiO_2_, and (c) the TiO_2_@MWCNTs nanocomposite.

Figure 3 shows TEM images of the MWCNTs, TiO_2_ powder, and the TiO_2_@MWCNTs nanocomposite. Figure 3a shows that the pristine MWCNTs are uniform and possess an external diameter of less than 50 nm with a wall thickness of roughly 10 nm. Moreover, the non-smooth walls of the MWCNTs indicates the presence of defects such as vacancies, dangling bonds, interstitials, and pentagons [14]. Figure 3b shows the irregular shape of TiO_2_ particles smaller than 20 nm and their non-uniform distribution. In Figure 3c, some MWCNTs link with TiO_2_ clusters as conjunctional bridges. TiO_2_ particles deposit only on the outside wall surface of the MWCNTs. Additionally, an agglomeration of TiO_2_ particles is only observed at the branching points, zigzag regions, and the end of MWCNTs where the defects are identified. However, the observation differs from previous studies, in which the TiO_2_ particles are uniformly attached to CNTs by layer-by-layer coating or sol–gel methods [15–17]. Notably, the defects on the wall surface of MWCNTs, which enable π–π interactions, could be the active sites to generate the TiO_2_ agglomerations via hydroxy groups and, thus, enhance the photoelectrochemical activity in aqueous environment [18–20].

**Figure 4:**
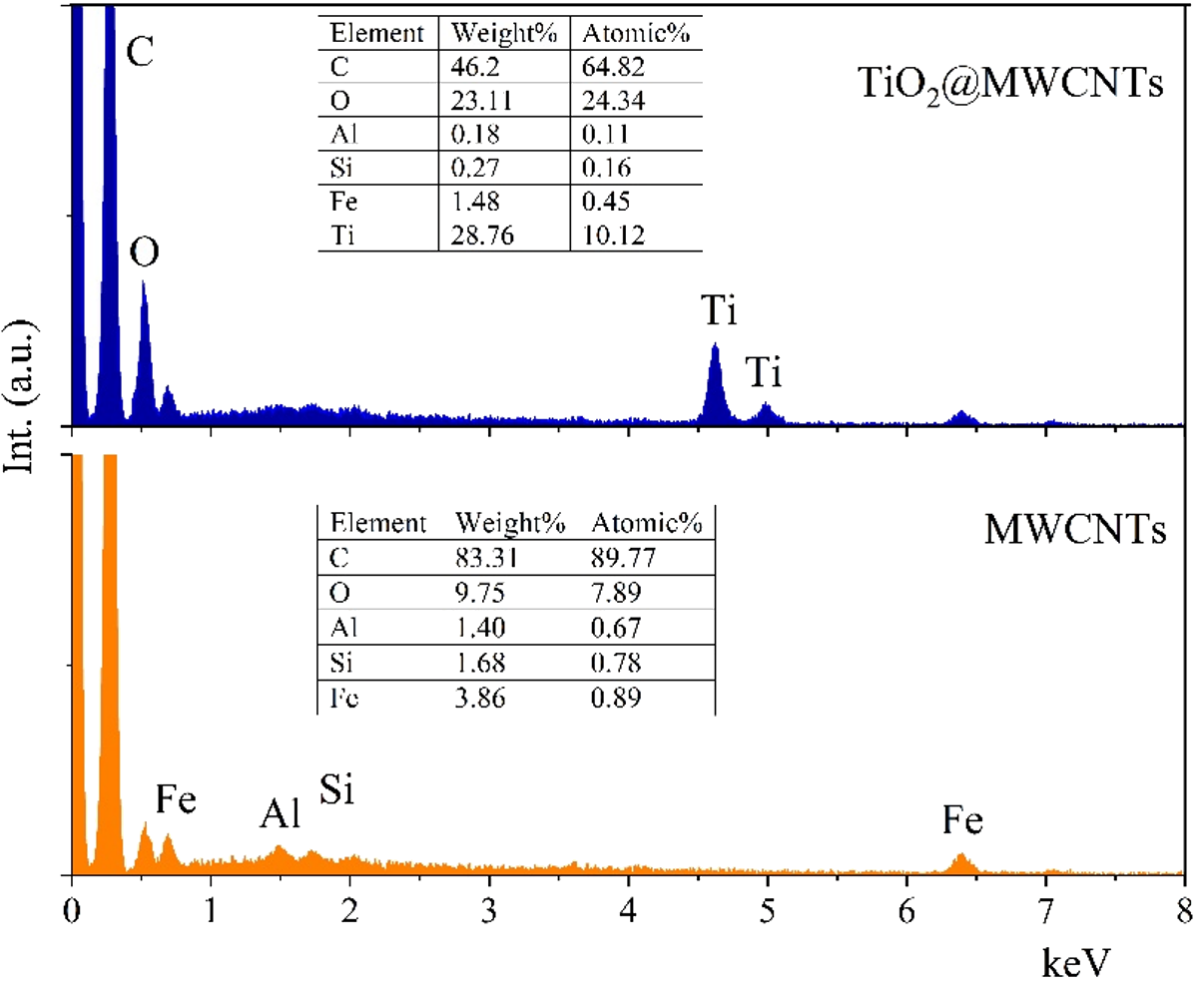
EDX spectra of MWCNTs and TiO_2_@MWCNTs.

Figure 4 shows the EDX spectra of MWCNTs and the TiO_2_@MWCNTs nanocomposite. The EDX spectrum for TiO_2_@MWCNTs confirms the presence of Ti, which accounts for 28.76 wt %. Small amounts of Fe, Al, and Si exists in as-synthesized MWCNTs and TiO_2_@MWCNTs, which could result from the catalyzed synthesis of MWCNTs [14].

**Figure 5:**
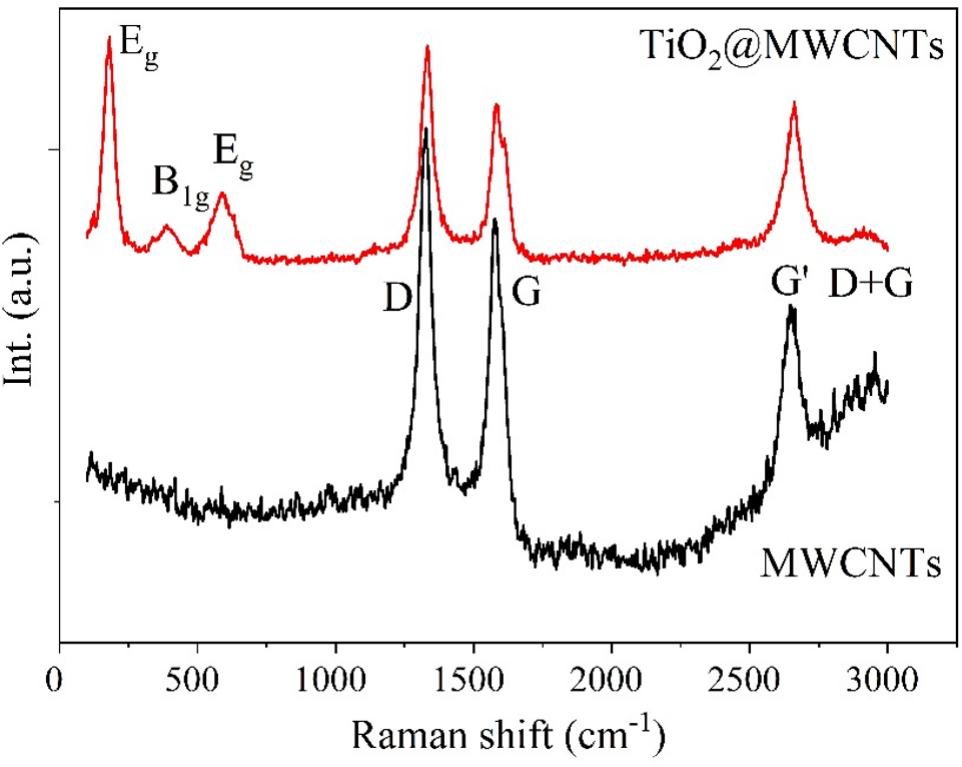
Raman spectra of MWCNTs and TiO_2_@MWCNTs.

Raman spectroscopy is applied for phase characterization of MWCNTs and TiO_2_@MWCNTS, as shown in Figure 5. The peaks at 178, 424, and 609 cm^−1^ are characteristic of the TiO_2_ phase in the TiO_2_@MWCNTs catalyst [21]. In the Raman spectrum of MWCNTs, there are two bands, that is, the D band at 1324 cm^−1^ and the G band at 1585 cm^−1^, which are ascribed to the defect structure and the ordered graphitic structure of the MWCNTs, respectively. The ratio between the D band and G band intensities (*I*_D_/*I*_G_) of the TiO_2_@MWCNTs catalyst is 1.45, higher than that for MWCNTs with 1.23. This observation indicates that TiO_2_ is a functional group on the outside wall of MWCNTs [22]. The ratios of *I*_G’_/*I*_D_ and *I*_G’_/*I*_G_ of TiO_2_@MWCNTs are 0.67 and 0.93, respectively. These ratios are higher than that for MWCNTs, with 0.41 and 0.51, respectively. The results reveal that TiO_2_ contributes to an increased number of defects on TiO_2_@MWCNTs [20]. Nevertheless, the intensities of the G bands belonging to the MWCNTs and the TiO_2_@MWCNTs nanocomposite, ascribed to the structure of the MWCNTs, are only slightly different. This reveals that the defects in the initial MWCNTs are hardly affected by the TiO_2_ nanoparticles. The TEM image also confirms that TiO_2_ nanoparticles only attach to some defects on the MWCNTs (Figure 3c) [17].

**Figure 6:**
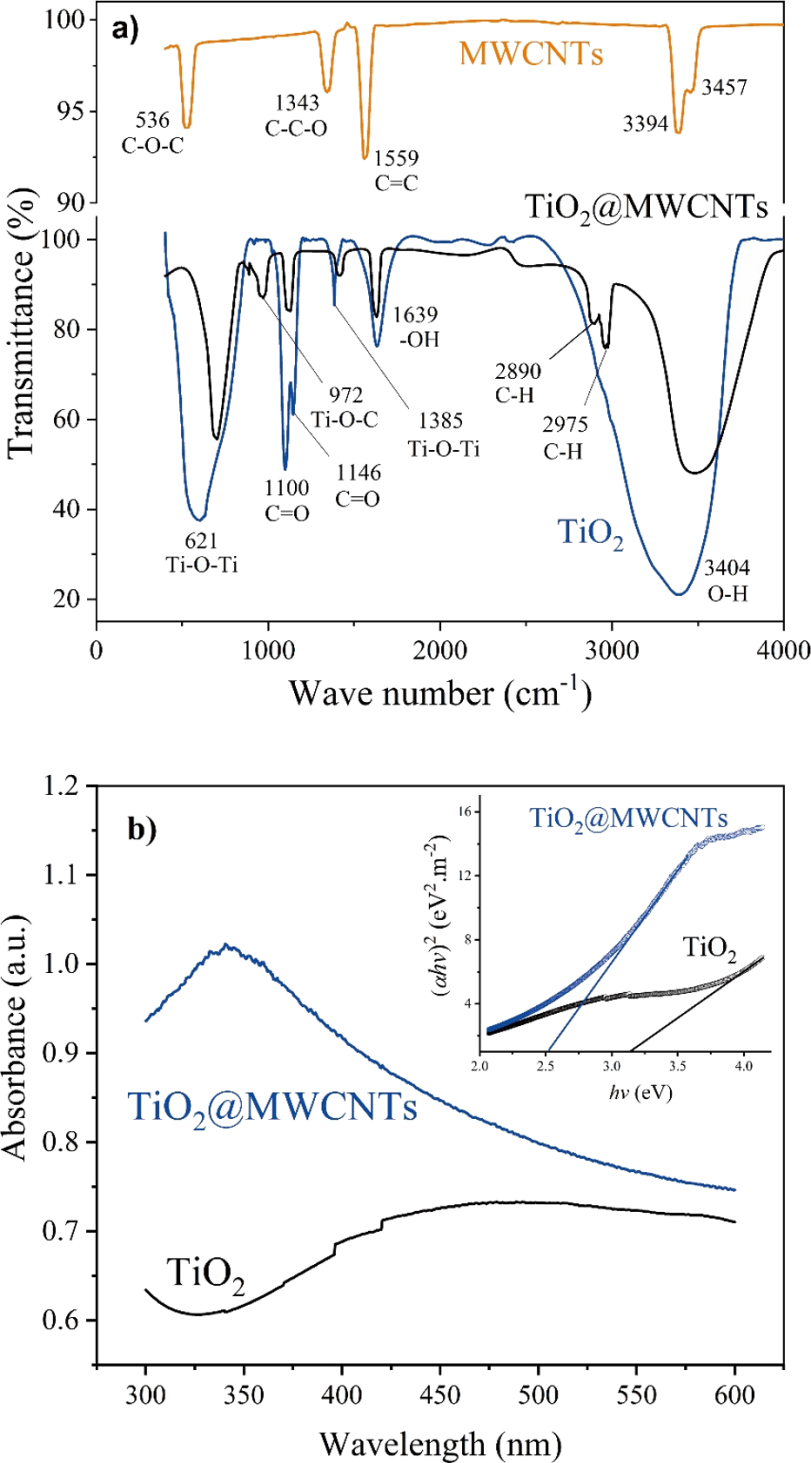
FTIR spectra of (a) MWCNTs, TiO_2_, and TiO_2_@MWCNTs, and (b) UV–vis DRS of TiO_2_ and TiO_2_@MWCNTs (Inset: Tauc plots).

FTIR spectra of MWCNTs, TiO_2_, and the TiO_2_@MWCNTs nanocomposite are shown in Figure 6a. Regarding the spectrum of MWCNTs, a typical peak at 1559 cm^−1^ is attributed to the vibration of C=C groups, whereas the peaks at 536, 1343, and 3394 cm^−1^ correspond to the C–O–C, C–C–O, and OH groups, respectively, on the MWCNTs [23]. For TiO_2_, a broad peak at 3404 cm^−1^ is attributed to the OH stretching, and another broad peak at 621 cm^−1^ is assigned to the Ti–O and Ti–O–Ti stretching of TiO_2_ [24]. In addition, the spectrum of TiO_2_@MWCNTs shows the characteristic peak at 972 cm^−1^ of the vibration of Ti–O–C groups, indicating the formation of a covalent bond between TiO_2_ and MWCNTs [24].

The UV–vis diffuse reflectance spectra of the prepared catalysts are shown in Figure 6b. The optical absorption of TiO_2_ is in the UV region, while the light absorption edge of TiO_2_@MWCNTs redshifts to the visible-light region. As seen from the Tauc plots (inset of Figure 6b), the optical band gap of TiO_2_ and TiO_2_@MWCNTs catalysts are calculated as 3.14 and 2.51 eV, respectively. The results show that the lower bandgap of TiO_2_@MWCNTs catalyst could derive from vacancies on the MWCNTs or the interaction between TiO_2_ and MWCNTs [25]. Moreover, the C–O–Ti linkages on the TiO_2_@MWCNs contribute to the extension of the absorption of light at a longer wavelength [26]. Accordingly, the low bandgap of TiO_2_@MWCNTs indicates improved visible-light absorption.

**Figure 7:**
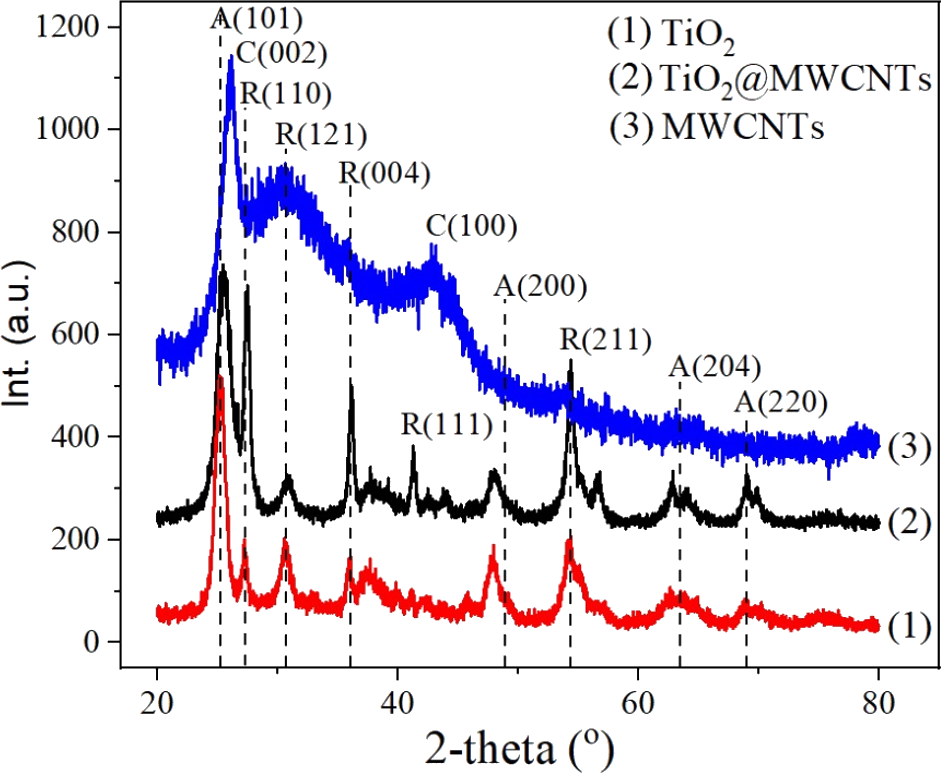
XRD patterns of MWCNTs, TiO_2_ and TiO_2_@MWCNTs nanocomposite.

XRD analysis is performed to confirm the crystalline structure and phase composition of TiO_2_, MWCNTs, and the TiO_2_@MWCNTs nanocomposite as described in Figure 7. Diffraction peaks at 26.1° and 42.6° correspond to the *d*-spacing between graphene sheets and the lateral correlation of graphite layers, which is presentative for MWCNTs [27]. Additionally, the XRD pattern of TiO_2_ exhibits peaks at 25.4° and 48.2°, ascribed to the anatase phase, while the other peaks at 27.6° and 36.2° are attributed to the rutile phase [28]. The weight fraction of anatase/rutile (*f*) relating to the intensity of the most substantial peaks (25.4° for anatase (*I*_A_) and 27.6° for rutile (*I*_R_)) is calculated to be 73.8 % using the estimated model *f* = 1/(1 + 1.26*I*_R_/*I*_A_) [29]. The overlap of the prominent peak at 26.1° for MWCNTs with that at 25.4° for anatase TiO_2_ results in a problematic identification for each component. Moreover, the rutile phase increases 3.5 times based on the intensity of the primary diffraction peak at 27.6° of TiO_2_@MWCNTs compared to that of TiO_2_. The observation indicates that anatase TiO_2_ transforms into rutile. This could be due to the carbon components on the MWCNTs acting as a robust reducing agent for facilitating the transformation from anatase to rutile TiO_2_ by forming oxygen vacancies [30].

**Figure 8:**
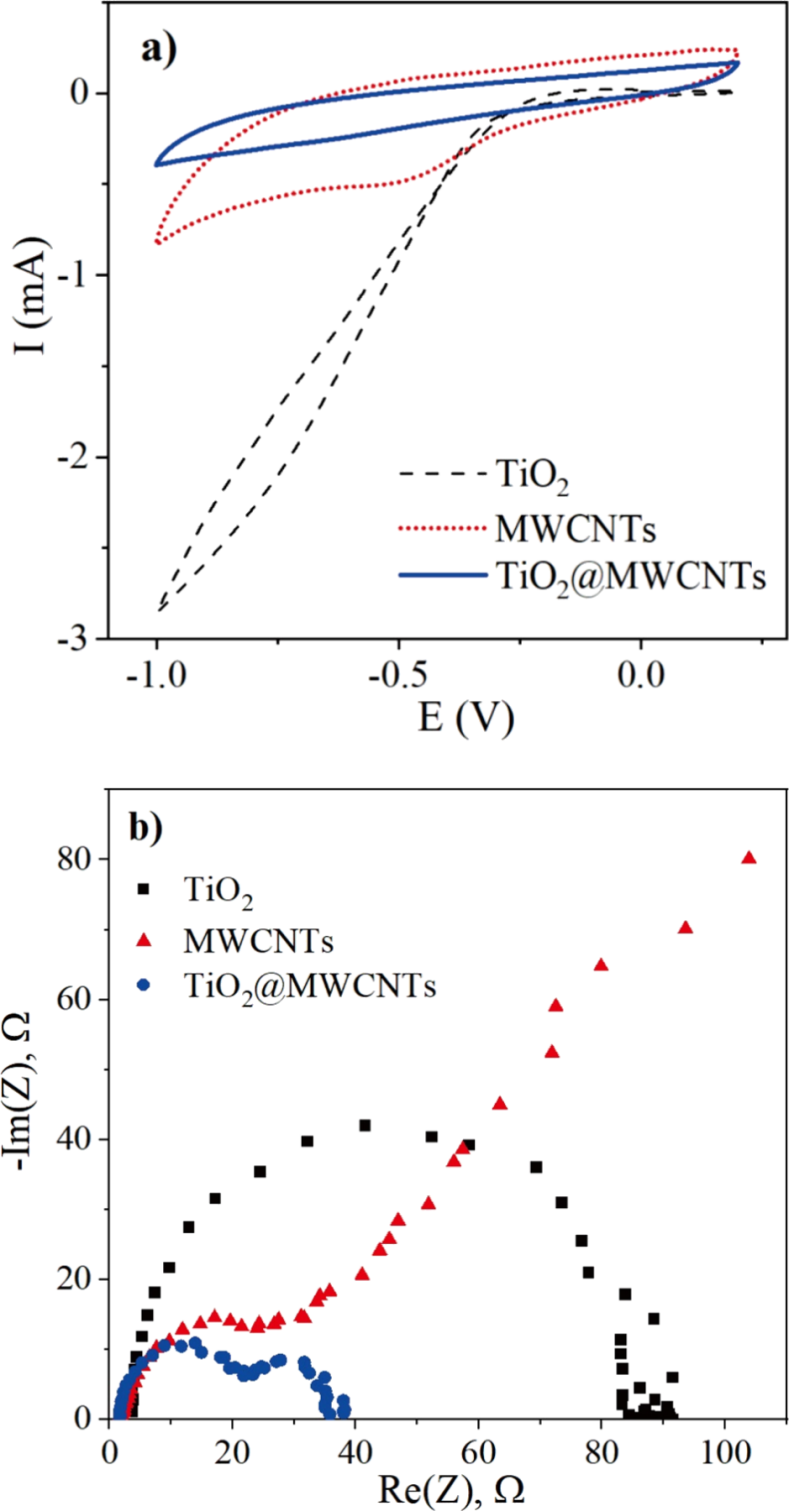
(a) Cyclic voltammograms in 0.1 M KCl at 50 mV/s of scan rate, (b) Nyquist plots from EIS measurements in 0.1 M KCl.

Generally, the capacitance of the photoelectrochemical electrode is associated with the photoelectrochemical processes occurring at the interface between electrode and electrolyte [31]. Cyclic voltammetry measurements are utilized to analyze the characteristics of charge and discharge of the photoelectrochemical electrodes. Figure 8a shows the cyclic voltammograms (CVs) generated using the prepared TiO_2_, MWCNTs, and TiO_2_@MWCNTs electrodes as working electrodes in 0.1 M KCl electrolyte at a sweep rate of 50 mV/s. In Figure 8a, oxidation and reduction peaks are not observed in the CVs in the scanned potential range from −1.0 to +0.2 V. In the CV of the TiO_2_ electrode, the current decreases significantly at a potential below −0.3 V, which could be due to the electron trap energy [32]. Moreover, the width of the CV for the MWCNTs electrode is more larger than that for TiO_2_ and TiO_2_@MWCNTs electrodes, indicating that the MWCNTs electrode possesses a porous surface and high capacitance derived from a thick electrical double layer (EDL) [33]. However, incorporating TiO_2_ onto the MWCNTs leads to a decrease of the EDL, increasing the electron transfer rate in the TiO_2_@MWCNTs electrode [34]. Puthirath et al. proved that the EDL has a significant influence on the hydrogen evolution reaction of the electrode [35]. Based on the cyclic voltammetry results, it could be suggested that the TiO_2_@MWCNTs electrode is superior regarding photoelectrochemical application compared to TiO_2_ and MWCNTs electrodes.

Electrochemical impedance spectroscopy (EIS) is applied to characterize the electron-transfer property of the electrodes through Nyquist plots, as shown in Figure 8b. The MWCNTs electrode has the lowest arc radius among the prepared electrodes, indicating the fast charge transport on this electrode [36]. A contrastive result is observed for the TiO_2_ electrode, which could be due to the poor electrical conductivity of TiO_2_ [37].

Table 1 shows the EIS parameters obtained from fitting the measured results with equivalent circuits. The *R*_1_ values illustrate a low electrical resistance of the 0.1 M KCl solution, while the *R*_2_ values show that the TiO_2_ electrode has the highest resistance among the prepared electrodes [38]. The results reveal a significant improvement in the electrical conductivity for the TiO_2_@MWCNTs electrode. The result agrees well with a previous study on TiO_2_@graphene composite electrodes [37]. Among the electrodes, the EDL capacitance (*C*_2_) at the TiO_2_ electrode surface is the lowest (1.32 μF), whereas that on the MWCNTs surface is the highest (29.64 μF). The EDL on the photoelectrochemical electrode surface contributes to the prevention of a fast carrier recombination and, hence, could improve the performance [39]. However, a thick EDL is detrimental to the photoelectrochemical performance because free electrons can shift to the trap state, resulting in a potential difference in the interface between the electrolyte and the electrode [40]. The Warburg element (W_3_) in the equilibrium circuit, indicating the contribution of diffusion to the overall charge transfer on the electrode, is not found in the TiO_2_ and TiO_2_@MWCNTs electrodes [41]. The EIS spectrum shows that the Voigt circuit is found to fit the TiO_2_ electrode. In contrast, the EIS spectrum of the TiO_2_@MWCNTs electrode, including two semicircle parts, demonstrates different responses of the electrode at low and high frequencies [42–43]. Additionally, the equivalent circuit fitted to the TiO_2_@MWCNTs electrode indicates that electron and ion transfers and the electrode material contribute to the overall charge transfer [43]. The results suggest synergies of TiO_2_ nanoparticles and MWCNTs in the TiO_2_@MWCNTs electrode.

**Table 1:** Parameters from fitting EIS results.

Electrode	Best fit circuit	Parameters	Values	Dev.

TiO_2_	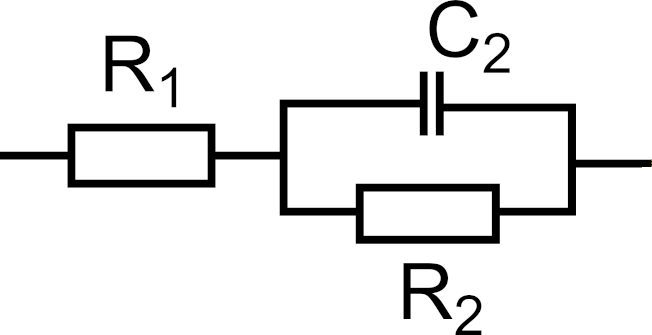	*R*_1_, Ω	3.457	0.2782
		*C*_2_, μF	1.324	27.18 × 10^−9^
		*R*_2_, Ω	84.04	0.3206

MWCNTs	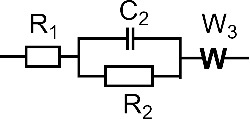	*R*_1_, Ω	1.385	0.2425
		*C*_2_, μF	29.64	2.971 × 10^−6^
		*R*_2_, Ω	17.84	0.3985
		W_3_, Ω	202	1.27

TiO_2_@MWCNTs	R_1_ + C_2_/(R_2_ + C_3_/R_3_)	*R*_1_, Ω	1.666	0.2652
		*C*_2_, μF	12.47	1.516 × 10^−6^
		*R*_2_, Ω	21.32	1.49
		*C*_3_, mF	0.493	0.175 × 10^−3^
		*R*_3_, Ω	13.78	1.661

### Effect of KOH concentration on photoelectrochemical water splitting

**Figure 9:**
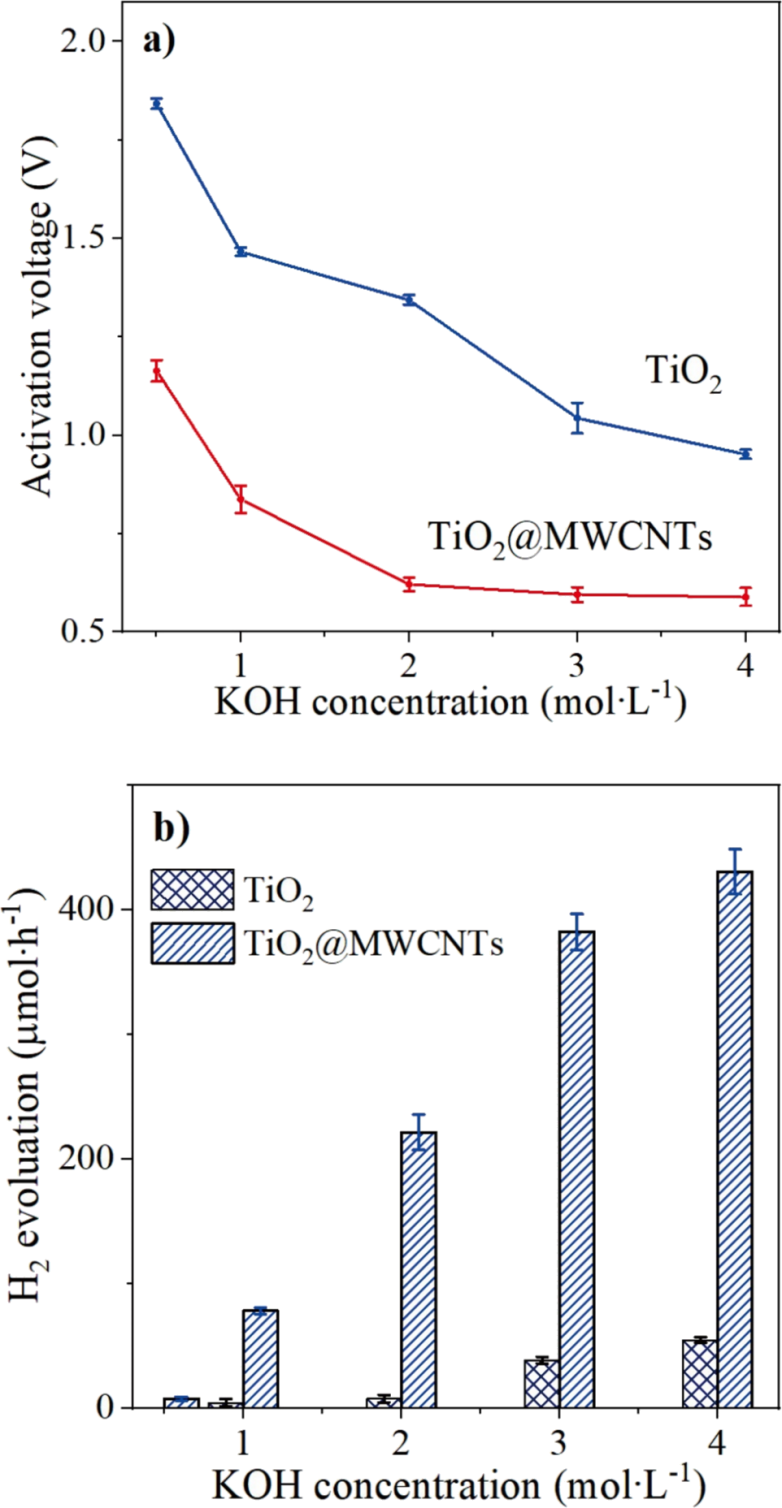
Effect of KOH concentration on (a) water splitting activation voltage and (b) rate of H_2_ evolution at a voltage of 1.0 V; measurements under sunlight with 85 ± 5 klux of illuminance.

Figure 9a shows the effect of KOH concentration on the activation voltage for the water redox reaction at the photoelectrochemical electrodes. The increase in KOH concentration leads to a decrease in the resistance of the electrolyte, thereby decreasing the activation voltage for water splitting or water redox reaction of the photoelectrochemical electrodes (Figure 9a) [44]. Notably, the activation voltage for water splitting of the TiO_2_@MWCNTs electrode is lower than that of the TiO_2_ electrode at all KOH concentrations. Furthermore, a KOH concentration higher than 3 M insignificantly affects the activation voltage of the TiO_2_@MWCNTs photoelectrochemical electrode.

The increase in KOH concentration could improve the electrical conductivity and the photocurrent of the photoelectrochemical electrode [44–45]. As seen in Figure 9b, the water splitting performance was significantly enhanced with increasing KOH concentration. However, the difference in the photoelectrochemical performance between KOH concentrations of 3 and 4 M is negligible. Furthermore, using high KOH concentrations for the electrolyte is not recommended to avoid corrosion of the electrodes [44]. Accordingly, it is suggested that the 3 M KOH electrolyte is suitable for the photoelectrochemical electrolyte.

### Photoelectrochemical behavior

**Figure 10:**
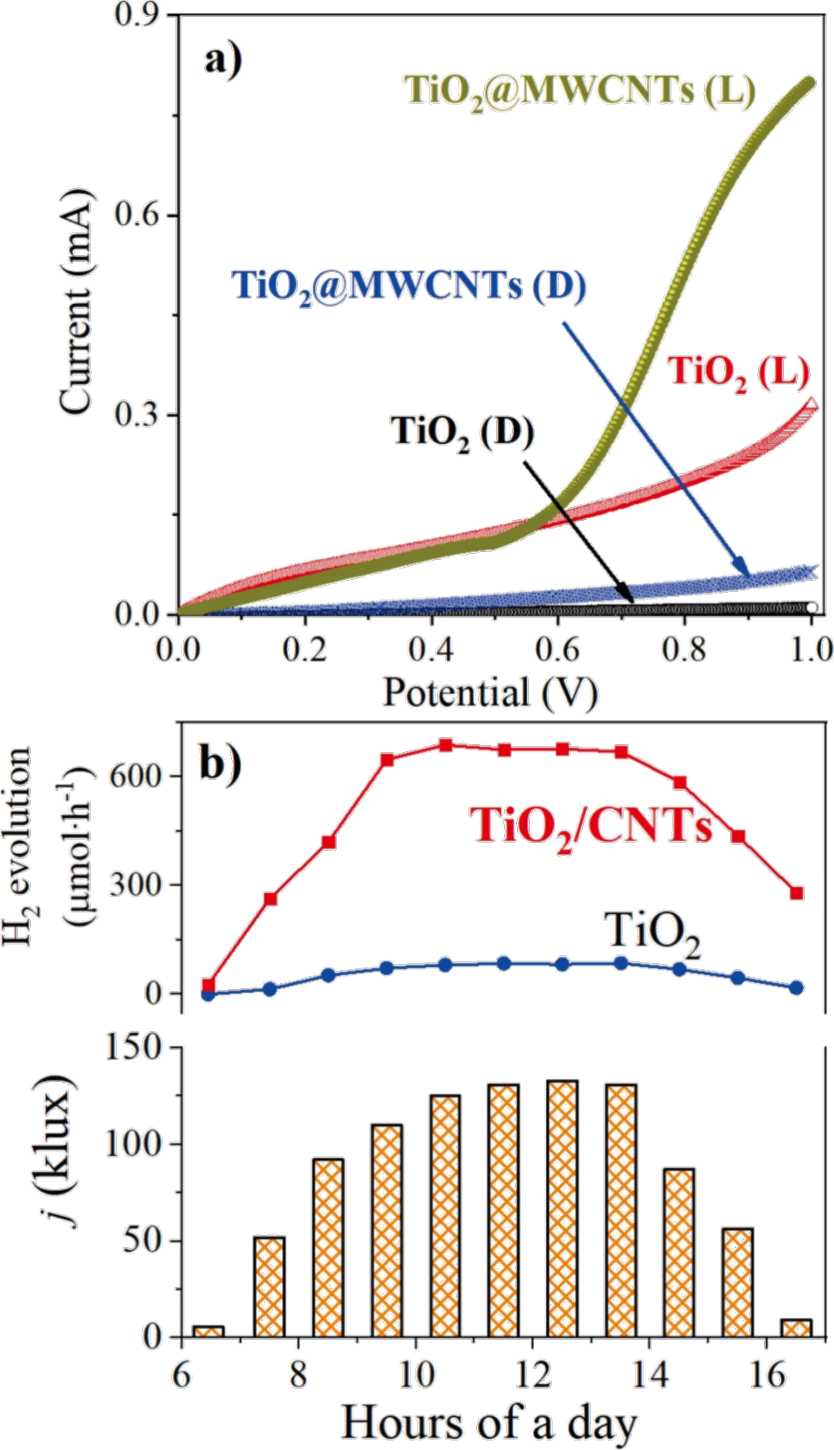
(a) Voltammograms of the TiO_2_ and the TiO_2_@MWCNTs electrode under dark (D) and light (L) conditions. (b) Dependence of luminous intensity and H_2_ evolution on the time of the day; electrolyte: 3 M KOH.

The relationships between the applied potential and the current density of TiO_2_ and TiO_2_@MWCNTs electrodes under dark (D) and light (L) conditions (luminous emittance values in the range of 20–40 and 50–60 klux, respectively) are shown in Figure 10a. The results show that the current density of both electrodes tested under light condition is more higher than that in the dark. The TiO_2_ electrode exhibits insignificant photocatalytic activity in the studied potential window. This confirms that the combination of TiO_2_ and MWCNTs enhances the visible-light absorption of the nanocomposite, even under weak light illuminance (dark conditions), which leads to the enhancement of the current density of the TiO_2_@MWCNTs electrode. At 1.0 V of voltage, the current density of the TiO_2_@MWCNTs electrode is about 30 times (D) and 10 times (L) higher than that of the TiO_2_ one. The results reveal that the TiO_2_@MWCNTs-based photoelectrochemical electrode is an effective photoelectrochemical catalyst under visible-light irradiation.

Figure 10b shows the hydrogen production and the average light intensity as a function of the time of the day from 6:00 AM to 5:00 PM. In Figure 10b, the sunlight illuminance peaks from 10:00 AM to 2:00 PM correlate with the highest hydrogen production of the TiO_2_@MWCNTs electrode. The TiO_2_ electrode exhibits poor hydrogen production under sunlight irradiation. It could be explained by the 3.14 eV optical band gap of TiO_2_, which absorbs only UV light. In addition, the fast recombination of the photogenerated (h^+^/e^−^) pairs contributes to the poor photochemical catalysis activity of the TiO_2_ electrode [46]. For the TiO_2_@MWCNTs nanocomposite, the TiO_2_ agglomerates attached to the MWCNTs are found to prevent the recombination of the h^+^/e^−^ pairs and the H^+^/O^−^ couples.

Furthermore, excitons in the MWCNT structure can absorb visible-light irradiation and accordingly produce a sub-cathode current [8], which significantly improves the rate of hydrogen evolution (Figure 10b). From 6:00 AM to 5:00 PM, the average rate of hydrogen evolution generated from the TiO_2_@MWCNTs electrode is 8.88 times higher than that from the TiO_2_ electrode. In particular, the feature increases 15 and 18 times at 7:00 AM and 4:00 PM, respectively, while identical sunlight spectra are revealed in the morning and in the afternoon [47]. The observation suggests that the photoactivity of the photoelectrochemical water splitting catalyst depends on photon energy and luminous emittance [9,46]. Furthermore, illumination higher than 100 klux solar allows the photoelectrochemical electrode to generate H_2_ at the highest rates.

The STH conversion efficiency is the ratio between the hydrogen production rate and the solar energy input [10–11]. Assuming a mean illuminance of the solar irradiation of 88.9% [48], the average STH conversion efficiency of the TiO_2_@MWCNTs electrode from 6 AM to 5 PM is 11.1%, as shown in Table 2. The photoelectrochemical water splitting performance of the TiO_2_@MWCNTs photoanode is superior compared to electrodes in previous studies [48].

**Table 2:** Photoelectrochemical water splitting performance of various TiO_2_-based catalysts.^a^

Photoanode (*E*_g_, eV)	Photocathode (*E*_g_, eV)	Light/source	Applied potential, V	STH conversion efficiency, %	n/p electrolyte	Ref. (year)

TiO_2_-SWCNTs	SCE	UV–vis/150 W Xe lamp	0	0.2	1 M KOH	[12] (2007)
TiO_2_ (3 eV)	GaP (2.35 eV)	UV–vis/450 W Xe lamp	0.96	0.25	0.2 N H_2_SO_4_	[49] (1976)
TiO_2_	Cu-Ti-O NT	UV–vis/300 W Xe lamp	0.3	0.30	0.1 M KOH/0.1 M NaHPO_4_	[50] (2008)
TiO_2_	np^+^Si	UV–vis/Xe lamp	0.0	0.39	1.0 M KOH/1.0 M H_2_SO_4_	[51] (2015)
C doped TiO_2_ NTs (2.75 eV)	RHE	vis/150 W Xe lamp	1.5	0.42	1.0 M KCl	[52] (2019)
Ag/TiO_2_/CNTs (2.8 eV)	Ag/AgCl	UV–vis/20 W·cm^−2^	1.0	4.4	0.5 M H_2_SO_4_	[53] (2016)
TiO_2_@MWCNTs (2.5 eV)	Cu	sunlight	1.0	11.1	3 M KOH	this work

^a^NTs: nanotubes; SCE: saturated calomel electrode; RHE: reversible hydrogen electrode; CNTs: carbon nanotubes; SWCNTs: single-wall CNTs; MWCNTs: multi-wall CNTs.

## Conclusion

A TiO_2_@MWCNTs nanocomposite photocatalyst is successfully synthesized via a simple hydrolysis method to fabricate the photoanode for photoelectrochemical water splitting. The TiO_2_@MWCNTs nanocomposite with a bandgap of 2.5 eV enables visible-light absorption of the electrode. Additionally, the coupling of TiO_2_ and MWCNTs hinders the recombination of photogenerated h^+^/e^−^ pairs and, thus, improves the hydrogen evolution of the electrode. The average rate of hydrogen evolution of the TiO_2_@MWCNTs electrode is 8.88 times higher than that of a TiO_2_ electrode operating under sunlight illuminance from 6 AM to 5 PM, demonstrating the superior photoelectrochemical performance of the TiO_2_@MWCNTs electrode for water splitting.
